# Extracorporeal membrane oxygenation in Non-Immune Hydrops fetalis – a registry-based data analysis over the last 25 years

**DOI:** 10.1007/s00431-026-07185-x

**Published:** 2026-06-26

**Authors:** Manuel Kuelshammer, Anna-Sophie Matzik, Andreas Hansmann, Johanna Moersdorf, Brigitte Strizek, Till Dresbach, Joachim Schmitt, Hemmen Sabir, Andreas Mueller, Lukas Schroeder

**Affiliations:** 1 Department of Neonatology and Pediatric Intensive Care Medicine, University of Bonn, University Hospital Bonn, Bonn, Germany; 2 Department of Obstetrics and Prenatal Medicine, University of Bonn, University Hospital Bonn, Bonn, Germany; 3 Center for Rare Diseases, University Hospital Bonn, University of Bonn, Bonn, Germany

**Keywords:** Non-immune hydrops fetalis, ECMO, Neonates

## Abstract

Non-immune hydrops fetalis (NIHF) is a rare condition that can lead to serious cardiopulmonary compromise after birth, often resulting in the need for intensive cardiorespiratory support, and in selected cases including extracorporeal membrane oxygenation (ECMO). Outcome is complicated due to a high rate of preterm birth. Morbidity and mortality data regarding NIHF and outcome of ECMO treatment in this population are very sparse. This is the first update and analysis of the Extracorporeal Life Support Organization (ELSO) registry-based data of neonates with NIHF. Neonatal patients with a diagnosis of NIHF and treated with ECMO during the years 2000–2024, reported to the ELSO registry, were retrospectively analyzed. Exclusion criteria were HF due to hemolytic disease, congenital diaphragmatic hernia (CDH), and major cardiac defects. After the application of exclusion criteria, 171 patients remained for final analysis, of these 88 (52%) survived until discharge. Infants with a higher birth weight (*p* = .011) and those with a gestational age (GA) ≥ 35 weeks (*p* = .032), as well as female patients (*p* = .027), had significantly higher in-hospital survival rates. The ECMO support type (veno-arterial vs. veno-venous) did not influence in-hospital mortality rate (*p* = .524) or complication rate. Female sex and higher BW (> 2.5 kg) remained independently associated with higher in-hospital survival after a binary logistic regression analysis.

*Conclusion*: Mortality rates over the last 25 years remained largely unchanged. Our analysis revealed baseline characteristics as independent predictors for higher in-hospital survival in these patients. In comparison with other underlying conditions treated with neonatal ECMO, neonates with NIHF present comparable morbidity and mortality rates after veno-arterial or veno-venous ECMO treatment.

**Table:** 

**What is Known:** • *NIHF is a rare neonatal disease leading to preterm birth and a high risk of cardiorespiratory compromise, sometimes resulting in the need for ECMO treatment*. • *ECMO treatment for preterm infants is feasible, with increasing survival rates*.	
**What is New:** • *After ECMO treatment, neonates with NIHF show acceptable complication rates and in-hospital survival, supporting a case-selective discussion of ECMO therapy*. • *Both veno-arterial and veno-venous ECMO are potential rescue therapy options in experienced neonatal ECMO centers with comparable outcomes*.

**What is Known:**

• *NIHF is a rare neonatal disease leading to preterm birth and a high risk of cardiorespiratory compromise, sometimes resulting in the need for ECMO treatment*.

• *ECMO treatment for preterm infants is feasible, with increasing survival rates*.

**What is New:**

• *After ECMO treatment, neonates with NIHF show acceptable complication rates and in-hospital survival, supporting a case-selective discussion of ECMO therapy*.

• *Both veno-arterial and veno-venous ECMO are potential rescue therapy options in experienced neonatal ECMO centers with comparable outcomes*.

## Introduction

Non-immune hydrops fetalis (NIHF) is a rare and severe intrauterine complication, with a prevalence of 1/1600 to 1/7000 pregnancies or 1/2500 to 1/3500 neonates [1]. It should be regarded as a common symptom of different underlying diseases with a wide variety of pathophysiological causes. Since the introduction of prophylaxis of the immunologically mediated hydrops fetalis due to Rhesus incompatibility (IHF), incidences of IHF have declined in developed countries, resulting in a higher portion for NIHF (80–85%) [2].

Pathophysiological mechanisms leading to NIHF can be multifactorial, and underlying causes range from genetic disorders, structural organ malformations, intrauterine infections, cardiovascular compromise through prenatal arrhythmias, non-compaction cardiomyopathies, twin-twin-transfusion syndrome (TTTS), metabolic disorders, hematologic disorders, and others [3–5]. In about 20–25% of the cases, the underlying cause remains unclear [1, 6, 7]. A frequent problem in pregnancies complicated by NIHF and IHF is preterm birth. In a prospective study with 56 cases of NIHF, preterm birth occurred in 86% of the cases, with a median gestational age of 33 weeks (GA) [8].

Innovative postnatal treatment includes respiratory support, vasoactive as well as anti-pulmonary hypertension treatment, drainage of effusions, therapy specific to the underlying cause (e.g., antiviral, antiarrhythmic, or antiproliferative therapy), targeted transfusions, reconstructive surgery (in case of congenital diaphragmatic hernia (CDH) or cystic pulmonary airway malformation or others). If conservative treatment fails, extracorporeal life support (ECLS) through veno-arterial (VA) or veno-venous (VV) extracorporeal membrane oxygenation (ECMO) has become a rescue therapy for these cases. ECMO can be performed via surgical vessel cannulation or single-/multistage percutaneous cannulation. However, due to the aforementioned high rate of prematurity, associated with higher mortality and a high risk of intracranial hemorrhage (ICH) as well as difficult vascular access (complicated through skin edema), it is an approach that requires extensive experience with this technique, often only provided in quaternary referral ECMO centers. There are only a few published case reports or cohort studies/case series related to neonates with NIHF [9, 10]. Registry-based data from the Extracorporeal Life Support Organization (ELSO) over the last decades are highly needed, as neonatal ECMO programs, neonatal ECMO circuits, and opportunities improved over the last decades. Therefore, the aim of this registry-based retrospective study is to give an update on ELSO registry–based data to VA- or VV-ECMO treatment for neonates with NIHF over the last 25 years. Besides, we want to give insights into a case series of neonates suffering from NIHF and treated with percutaneous VV-ECMO in our referral ECMO center.

## Material and methods

### Study design and participants

For this retrospective cohort study, we analyzed data from the ELSO registry for neonates treated with VA- or VV-ECMO in the period of 2000–2024 with a primary or secondary diagnosis of NIHF (ICD 9 code 778.0 and ICD 10 codes P83.2, P83.3, P83.30, and P83.39).

Exclusion criteria were as follows: IHF, CDH, and major cardiac defects (as per the definition of the guidelines of the American College of Cardiology/American Heart Association [11]). Neonates with minor cardiac defects (e.g., ventricular septal defect, atrial septal defect, patent ductus arteriosus, pulmonary stenosis or insufficiency, dextro- or levocardia, persistence of the left upper vena cava) as well as inherited rhythm disorders, such as congenital heart block, and malformations of the vessels, such as arteriovenous malformations, were included in the analysis.

### Patients consent and ethical approval

The study was approved by the local ethics committee of the Medical Center of the University of Bonn (local running number 2026–262-BO). Written informed consent was waived due to the retrospective design of the study. The methods used for the clinical research were performed in accordance with the STROBE (strengthening the reporting of observational studies in epidemiology) guidelines [12] and in accordance with the Declaration of Helsinki.

### Variables and setting

Demographic data included sex, birth weight (BW) and length, age at the start of ECMO treatment, GA in weeks post menstrual age, APGAR score at 5 min, year of treatment, pre-ECMO cardiac arrest, ventilation type prior to ECMO treatment, and ECMO mode (VA, VV, or other).

The outcome data included the duration of ECMO treatment, reason for discontinuation of ECMO, in-hospital mortality, and complication rates. Subgroup analyses for survivors and non-survivors, VA- and VV-ECMO-treated neonates, and patients < 35 and ≥ 35 weeks GA (below and above median GA) were performed.

Cardiovascular complications included the following outcomes: need for inotropic support, pulmonary hypertension (PH), arterial hypertension, cardiac arrhythmias, cardiac tamponade, myocardial stun, or need for cardiopulmonary resuscitation. Pulmonary complications included the following outcomes: pneumothorax and pulmonary hemorrhage. Renal complications were regarded as renal failure or requirement of renal replacement therapy (RRT). Neurologic complications included CNS hemorrhage, infarction, or ischemia, seizures, and brain death. Hemorrhagic complications included disseminated intravascular coagulation, bleeding on cannulation or surgical site, severe hemolysis, or gastrointestinal hemorrhage. Furthermore, metabolic complications included hyper- or hypoglycemia, hyperbilirubinemia, moderate hemolysis, acidosis, and alkalosis. Infectious complications were composed of culture-proven sepsis and leukopenia, and mechanical complications included thrombosis or clots, cannula problems, pump or oxygenator failure, air in circuit, cracks in pigtail connectors, or the need for circuit change. All complications were defined according to the ELSO registry data definition form.

### Statistical analysis and power analysis

As no comparable data sets for outcome data of neonates with NIHF and ECMO treatment were available, we did not perform a power calculation. With 171 included patients and an overall survival rate of approximately 52%, the study has sufficient power to detect moderate-to-large differences between balanced comparison groups. For an approximately balanced comparison of survivors and non-survivors, the available sample size would allow detection of an effect size of approximately Cohen’s *d* = 0.43 with 80% power at a two-sided alpha level of 0.05. Survival to discharge was defined as the primary outcome. The following parameters were defined as secondary outcomes: duration of VA- or VV-ECMO treatment, and complication rate (cardiovascular, pulmonary, renal, neurologic, metabolic, hemorrhagic, infectious, and mechanical complications).

Descriptive statistics were analyzed regarding patient demographics, pre-ECMO factors, and postnatal parameters. Normally distributed data are displayed as mean with standard deviation (± SD) and non-normally distributed data are displayed as median with inter-quartile range (IQR). For comparison of continuous and non-normally distributed variables, a Mann–Whitney *U* test was performed to compare continuous variables between two groups and subgroups. For categorical variables, the Pearson’s chi-square test was applied. Variables significantly associated with in-hospital survival in the univariate analysis (GA, BW, sex, and ICH during ECMO) were incorporated in a binary logistic regression model analyzing factors independently associated with a favorable outcome. A *p*-value of < 0.05 was considered significant. Statistical analysis was carried out using statistical software (IBM SPSS Statistics for Windows, Version 31.0, IBM Corp, Armonk, New York).

### Results

**Fig. 1:**
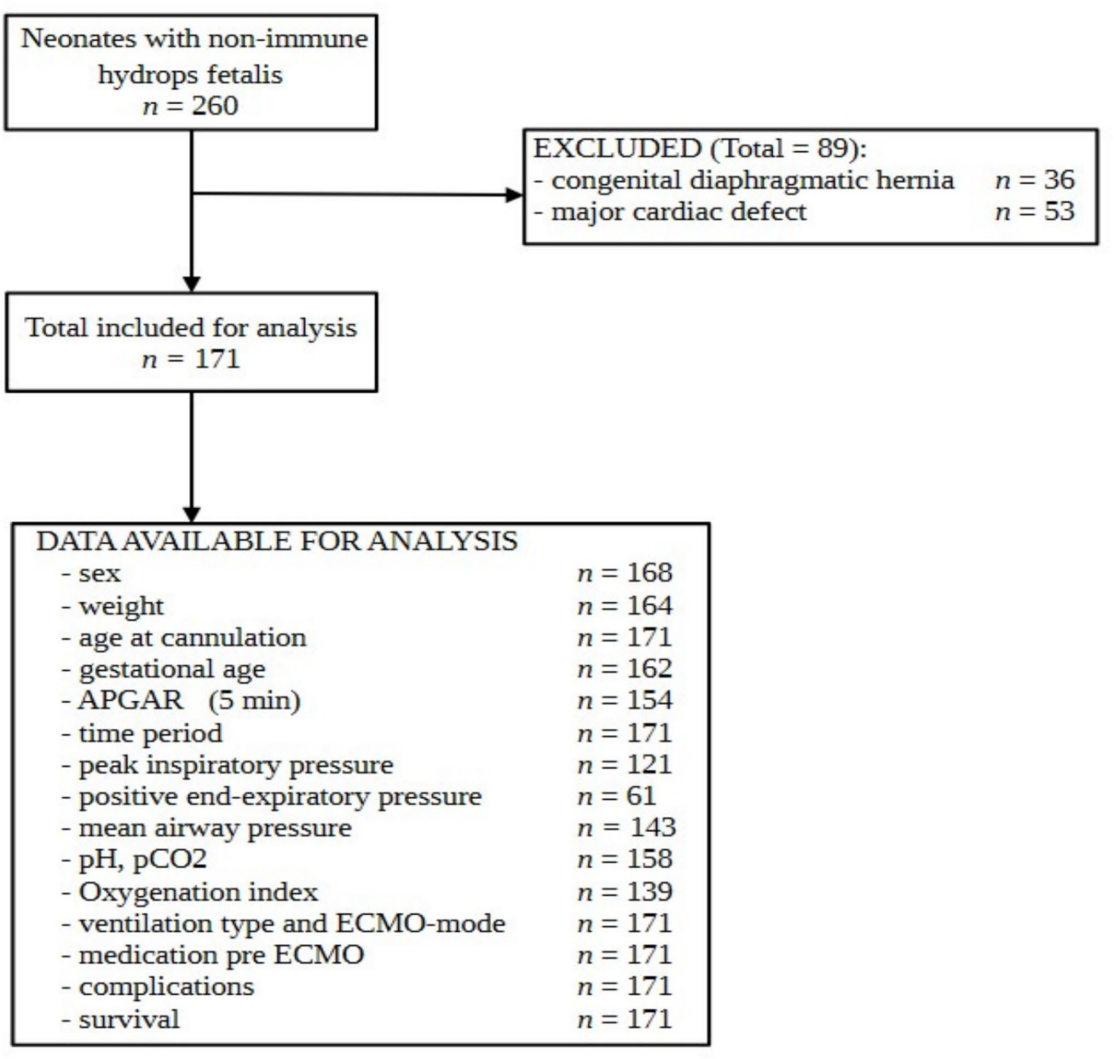
Flow-chart for study inclusion and exclusion

Overall, 260 neonates matched the inclusion criteria (NIHF). Of these, 89 neonates were excluded (36 neonates with CDH, and 53 neonates with an underlying major cardiac defect). After applying the exclusion criteria, 171 neonates remained for final analysis (see Fig. 1).

**Fig. 2:**
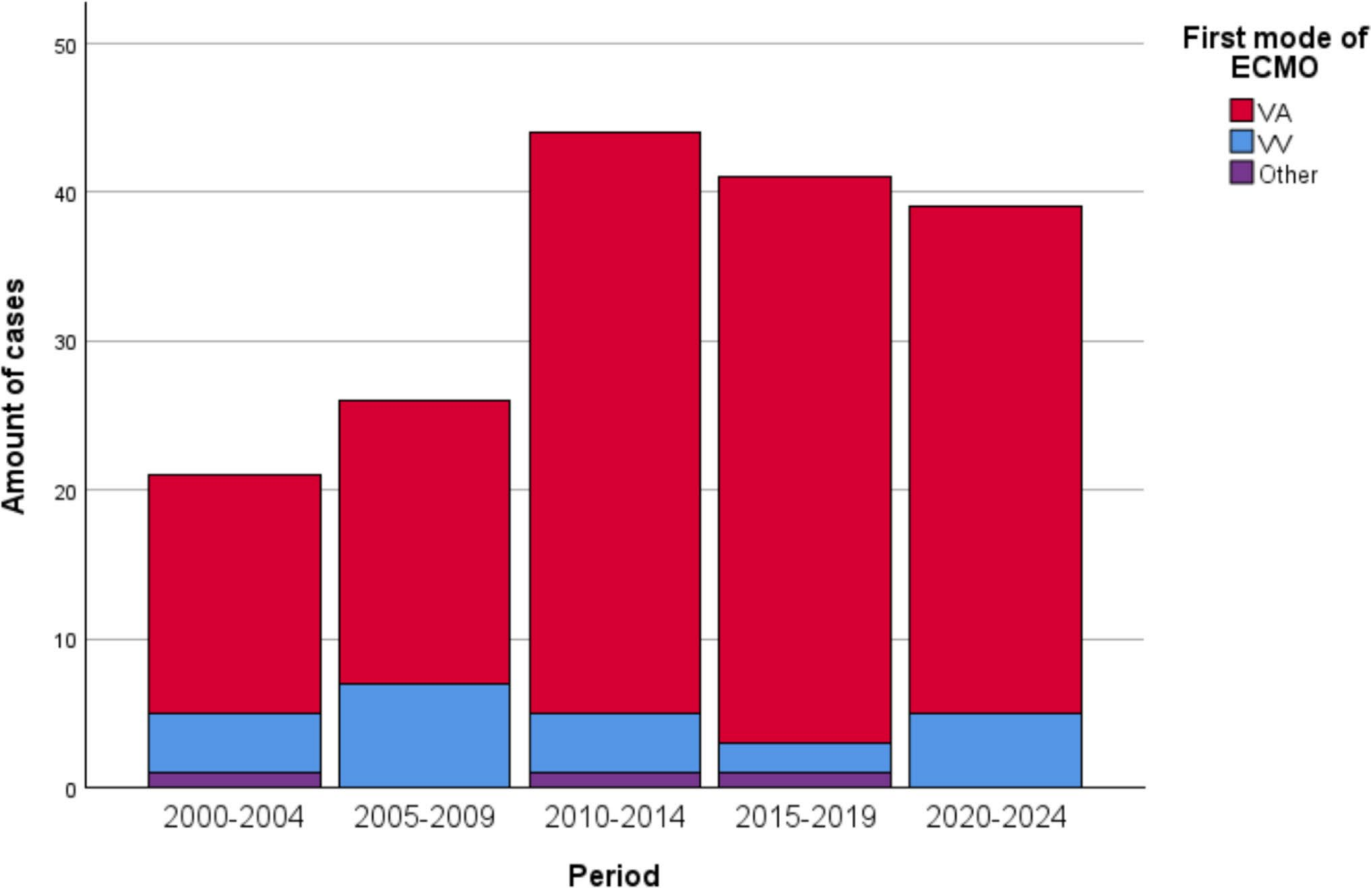
Total number of ECMO runs per 5-year period from 2000 to 2024. VA, veno-arterial ECMO; VV, veno-venous ECMO; others, veno-veno-arterial ECMO and unknown

Epidemiologic data are displayed in Table 1. Of the 171 neonates, 65 were female (38.7%), the median BW was 3 kg (2.6–3.5, IQR), and the median length was 48 cm (46–51, IQR). Overall, 68% of the cohort were born preterm (< 37 + 0 weeks of GA), and 39% of the neonates were born below 35 + 0 weeks of GA. APGAR score at 5 min was 6 (4–8, IQR). Numbers of treated neonates with VA- and VV-ECMO are displayed in Fig. 2. Comparing the time periods up to the years 2010–2014, the numbers of NIHF patients were rising steeply. However, after this period, registry data emphasize a slow decline in cases. VA-ECMO comprises the vast majority of cases (85%), followed by VV-ECMO (13%), and only one case of VVA-ECMO. In three of the 171 included cases, there was a conversion from VV- or VA-ECMO (not shown in the figures). Median duration of ECMO treatment was 160 h (99–267, IQR).

**Table 1:** Demographic and treatment data as well as complications in ECMO survivors and non-survivors’ data

Variables	Survivors (*n* = 88)	Non-survivors (*n* = 83)	*p*-value
Birth weight (kg)^#^	3.3 (2.7–3.55)	3.0 (2.52–3.25)	.011*
Sex (male), % (*n*)	53.4% (47)	70% (56)	**.027**^**+**^
Gestational age (< 35 w), % (*n*)	29% (26)	44% (37)	**.032**^**+**^
Gestational age (weeks)^#^	35.6 (34–38)	35 (34–37)	.041*
APGAR 5 min^#^	6.5 (4–8)	6 (2–6)	.924*
Age at cannulation (*d*)^#^	1 (1–3.5)	2 (1–8.5)	.296*
Pre-ECMO data			
Cardiac arrest, % (*n*)	18% (16)	20.5% (17)	.703^+^
Conventional invasive MV, % (*n*)	32% (27)	28.2% (20)	.132^+^
PIP (mbar)^#^	37 (28–44)	39 (29.5–46.5)	.628*
PEEP (mbar)^#^	6 (5.3–8)	8 (5.5–9)	.244*
Mean airway pressure (mbar)^#^	20 (16–22)	18 (15–21.8)	.456*
Oxygenation index^#^	49 (31–70)	47 (30–63)	.609*
pH^#^	7.16 (7.03–7.26)	7.16 (7.05–7.25)	.611*
pCO_2_ (mmHg)^#^	61.5 (43.5–85)	60.4 (45–70)	.280*
Lactate (mmol/L)^#^	3.9 (1.5–10.3)	3.4 (1.8–7.3)	.942*
Inotropes, % (*n*)	58% (51)	56.6% (47)	.861^+^
Vasopressors, % (*n*)	14.8% (13)	15.7% (13)	.871^+^
Inodilators, % (*n*)	21.6% (19)	14.5% (12)	.226^+^
Nitric oxide, % (*n*)	79.5% (70)	72.3% (60)	.267^+^
Complications during ECMO			
Pneumothorax, % (*n*)	9% (8)	7% (6)	.657^+^
Pulmonary hemorrhage, % (*n*)	5% (4)	10% (8)	.193^+^
Inotropes on ECMO, % (*n*)	27% (24)	33% (27)	.453^+^
Other cardiovascular complication, % (*n*)	12% (11)	19% (16)	.224^+^
Renal replacement therapy, % (*n*)	36% (32)	36% (30)	.976^+^
CNS hemorrhage, % (*n*)	11% (10)	25% (21)	**.018**^**+**^
CNS infarction, % (*n*)	2% (2)	4% (3)	.603^+^
Hemolysis, % (*n*)	9% (8)	7% (6)	.657^+^
Culture proven infection, % (*n*)	2% (2)	1% (1)	.595^+^
Leukopenia, % (*n*)	2% (2)	4% (3)	.603^+^
Mechanical complications, % (*n*)	34% (30)	46% (38)	.118^+^

Patient demographics, pre-ECMO parameters, and complication rates comparing survivors and non-survivors). Inotropes: dopamine, dobutamine, and epinephrine; vasopressors: norepinephrine, phenylephrine, and vasopressin; inodilators: milrinone and levosimendan; mechanical complications: thrombosis or clots, cannula problems, pump or oxygenator failure, air in circuit, cracks in pigtail connectors, or the need for circuit change

*CNS* central nervous system, *ECMO* extracorporeal membrane oxygenation, *MV* mechanical ventilation, *PEEP* positive end-expiratory pressure (mbar), *PIP* peak inspiratory pressure (mbar)

^*^Mann–Whitney *U* test

^+^Pearson’s chi-square

^#^Median (inter-quartile range)

**Fig. 3:**
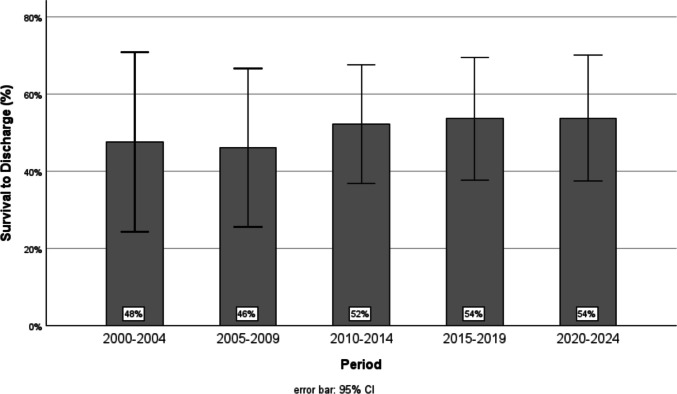
Total number of neonates with survival to discharge per 5-year period from 2000 to 2024

Overall survival to discharge was 52%. Outcome data after ECMO treatment in relation to treatment period are illustrated in Fig. 3. Overall, there are no significant differences in short-term outcome during recent years. Neonates surviving to discharge presented a significantly higher BW (3 vs. 3.3 kg, *p* = 0.011) and significantly more often female sex (63% vs. 46%, *p* = 0.027). Neonates with a higher GA (≥ 35 weeks of GA) presented significantly higher in-hospital survival rates (59% vs. 42%, *p* = 0.037). Subdividing the cohort in GA quartiles, no significant differences between subgroups regarding in-hospital survival were found (*p* = 0.13). In the lowest GA quartile (≤ 34 weeks GA), still a substantial portion (27%) of patients survived until discharge. Regarding other epidemiologic data and pre-ECMO parameters, we found no significant differences in survival (Table 1). There was also no significant difference in survival rates when comparing the initially applied ECMO modality, with an in-hospital survival of 53% in the VA-ECMO group vs. 46% in the VV-ECMO group (*p* = 0.524). Likewise, no significant differences between VA- and VV-ECMO groups were noted in relation to the duration of ECMO treatment (160 vs. 151 h, *p* = 0.717) and complications during ECMO treatment, as shown in Table 2.

**Table 2:** Comparison between initial ECMO modality (VA- and VV-ECMO)

Variables	VA-ECMO (*n* = 146)	VV-ECMO (*n* = 22)	*p*-value
Age (*d*)^#^	1 (1–3)	1 (1–2.25)	.771*
Birth weight (kg)^#^	3 (2.54–3.5)	3.1 (2.8–3.6)	.359*
Sex (male)	62.9% (90)	54.5% (12)	.451^+^
Gestational age (< 35 w)	42% (59)	20% (4)	.058^+^
Gestational age (weeks)^#^	35 (34–37.15)	36.15 (35.25–38)	.050*
APGAR 5 min^#^	6 (4–8)	7 (4.75–8)	.647*
Pre-ECMO data			
Cardiac arrest, % (*n*)	20.5% (30)	13.6% (3)	.447^+^
Conventional MV, % (*n*)	31.1% (41)	23.8% (5)	.519^+^
Discharged alive, % (*n*)	52.7% (77)	45.5% (10)	.524^+^
‍Complications during ECMO			
Pneumothorax, % (*n*)	7% (10)	14% (3)	.267^+^
Pulmonary hemorrhage, % (*n*)	6% (9)	9% (2)	.605^+^
Inotropes on ECMO, % (*n*)	29% (42)	36% (8)	.468^+^
Other cardiovascular complications, % (*n*)	16% (24)	9% (2)	.374^+^
Renal replacement therapy, % (*n*)	36% (52)	41% (9)	.630^+^
CNS hemorrhage, % (*n*)	19.2% (28)	14% (3)	.532^+^
CNS infarction, % (*n*)	3% (5)	0% (0)	.378^+^
Hemolysis, % (*n*)	8% (12)	9% (2)	.890^+^
Culture proven infection, % (*n*)	2% (3)	0% (0)	.497^+^
Leukopenia, % (*n*)	2% (3)	9% (3)	.070^+^
Mechanical complications, % (*n*)	40% (58)	36% (8)	.763^+^

Comparison between patients initially treated with VA- and VV-ECMO. Mechanical complications: thrombosis or clots, cannula problems, pump or oxygenator failure, air in circuit, cracks in pigtail connectors or the need for circuit change

*CNS* central nervous system, *ECMO* extracorporeal membrane oxygenation, *MV* mechanical ventilation

^*^Mann–Whitney *U* test

^+^Pearson’s chi-square

^#^Median (inter-quartile range)

Complications are listed in Tables 1 and 2. Regarding major complications, 30% of all neonates required inotropes, 2% required vasodilators due to arterial hypertension, 8% had a pneumothorax, 7% had pulmonary hemorrhage, 18% had an ICH, 2% had an intracranial infarction, and 36% required RRT. Neonates with a fatal outcome presented significantly higher incidences of ICH (25% vs. 11% in surviving neonates, *p* = 0.018). No other significant differences regarding separate complications could be found (see Table 1). Overall, there were no significant differences seen in complication rates between VA- and VV-ECMO-treated neonates (see Table 2).

After a binary logistic regression analysis, female sex (*p* = *0.0*28) and birth weight > 2.5 kg (*p* = *0.0*27) remained significantly associated with in-hospital survival, but not higher GA (> 35 weeks) or ICH.

In the period from 2022 to 2024, we treated five preterm neonates (GA, 32–35 weeks) with NIHF with VV-ECMO after percutaneous vessel cannulation with dual-lumen or multistage single-lumen cannula at our institution. The underlying etiology of NIHF in these cases was a congenital lymphatic malformation (CLM) in all neonates (5/5), with an underlying Noonan syndrome in three of the neonates and one neonate with an underlying trisomy 21. All received intensive vasoactive therapy, including dobutamine, milrinone, vasopressin, and noradrenaline, as well as nitric oxide. Four of five patients also received levosimendan prior to the beginning of ECMO. Time from intubation to ECMO start was 7 h median (range, 4–25 h). Survival was 80% with one fatal case of a preterm infant with a birth weight of < 2 kg who suffered a severe ICH during ECMO treatment.

## Discussion

The key findings of this retrospective cohort study of the ELSO registry can be summarized as follows: for neonates with NIHF, who are often prone to prematurity and at high risk for cardiopulmonary failure, ECMO can be a lifesaving treatment option. In-hospital survival was 51.5% in the present cohort, with better outcomes for female patients, neonates with a higher BW, and higher GA. Survival did not depend on the time period, initially applied ECMO mode or APGAR score, but was significantly lower in patients who developed ICH (*p* = *0.0*18). Female sex and higher BW (> 2.5 kg) were found independently associated with higher in-hospital survival after a binary logistic regression analysis incorporating GA, BW, sex, and ICH during ECMO. In the present cohort, there was no difference in complication rates between VA- and VV-ECMO-treated neonates.

### NIHF general outcome

General outcome of neonates with NIHF is linked to the etiology of the underlying disease and the maturity of the neonates. Neonates with a normal karyotype and no associated malformations present better outcomes [13]. Preterm birth in NIHF neonates resulted in a higher mortality rate (73% vs. 17% in term neonates). Reasons for preterm delivery included spontaneous labor (including premature rupture of membranes), new or worsening effusions, non-reassuring antenatal testing, and maternal indications, especially arterial hypertension [8]. Various factors led to a high rate of prematurity in pregnancies complicated by NIHF with a median GA of 32–33 weeks [6, 8, 13]. Reported long-term outcomes of neonates suffering from NIHF were widely varying and depended on GA at birth, polyhydramnios during pregnancy, and associated structural defects. In summary, a neonatal mortality of 35% and an overall mortality of 43% (448 out of 1037 patients) up to the age of 1 year was reported [14].

### ECMO in NIHF neonates in different time periods

During the last decades, the application of ECMO in neonatal patients has experienced a major change regarding indications, duration of ECMO treatment, and outcomes. Whereas meconium aspiration syndrome, respiratory distress syndrome, and sepsis comprised decreasing portions of ECMO-treated newborns, CDH, idiopathic persistent pulmonary hypertension of the newborn, and not otherwise specified respiratory failure increasingly became reasons for ECMO treatment. In the present data, we noticed a relatively steep rise of case numbers from the period 2000–2009 to 2010–2014, followed by a rather slow decline during recent years. This is in accordance with other reported data [15]. The recently declining numbers may be a sign of improving conservative treatment options. Survival of ECMO-treated neonates is highly dependent on the indication for ECMO initiation. Including all reported neonatal ECMO runs, declining survival rates have been reported (76% in 1994/95 and 67% in 2014/15) [16]. Recent data from the ELSO registry also reported a somewhat decreasing survival rate for neonates treated with ECMO due to respiratory failure (74% in the period 1989–2016, compared to 69% during the period 2009–2021) [17, 18]. Interpretation of these numbers is difficult due to differences in the patient profile, management strategies on ECMO, and potentially unknown factors. Average ECMO-run times have increased during the last years [19].

### Comparison with other ECMO indications

In term neonates with pulmonary hypoplasia, ECMO treatment has become a standard treatment in cases of cardiocirculatory or respiratory failure with overall increasingly good outcomes [15]. Patients with congenital renal and urological anomalies have an overall survival rate of 42%, whereas patients with a urogenital obstruction have a more favorable outcome than those with an intrinsic renal disease (survival to discharge 17% vs. 71%) [20]. In a single-center cohort study of neonates with congenital renal failure, survival to discharge after ECMO treatment was 29% [21]. For neonates with cardiac disease or extracorporeal cardiopulmonary resuscitation (ECPR), a survival of 48% and 44%, respectively, has been reported [18]. Another study reported a survival rate of 38% after ECPR [22]. In the spectrum of various indications for neonatal ECMO, patients with NIHF, as reported in this study, have a moderate outcome with an in-hospital survival of 52%.

### The role of prematurity and birth weight

In preterm neonates, outcomes after ECMO treatment have improved during the last decades with fewer intracranial bleeding events, fewer mechanical complications, and lower mortality [19, 23]. Regardless of the missing long-term data on neurologic development, recent reviews plead for considering ECMO treatment in preterm as early as 32 to 33 weeks GA with a BW < 2 kg [24]. In the present cohort, neonates with GA < 35 weeks at birth presented a significantly lower in-hospital survival after ECMO treatment compared to more mature neonates (*p* = 0.032). Still, about one-third of the patients survived to discharge with a GA < 34 weeks. Treatment decisions, therefore, will have to be made on an individual evaluation with close communication with parental guardians and patients’ families, even more in this highly vulnerable group. Our data emphasize that a higher BW at cannulation (> 2.5 kg) might have more impact on in-hospital survival than a higher GA (> 35 weeks) at cannulation. However, this data need to be interpreted with caution. BW in neonates with NIHF is biased by the edema itself and does not mirror the actual estimated and predicted BW. Nevertheless, higher BW might influence vascular size, and neonates with a higher BW might be easier to cannulate for ECMO.

### Comparing VA and VV

As in other reports, most patients (87% vs. 13%) were treated with VA-ECMO. Survival rates did not differ significantly with an in-hospital survival of 53% in the VA-ECMO and 46% in the VV-ECMO group (*p* = 0.524). Of note, VA-ECMO-treated neonates were almost significantly more premature than those treated by VV-ECMO (*p* = 0.05). However, when GA was considered, survival rates for both groups still did not differ significantly (*p* = 0.32).

### Complications during ECMO treatment

Complication rates during and after neonatal ECMO are known to be linked to the underlying disease and the patient group. Bagdure et al. [20] examined neonates with lung hypoplasia due to renal or urologic diseases and reported an incidence of 51% for RRT and 42% for a creatinine of 1.5–3 mg/dl. In children with ECPR, the incidence of RRT was 36%, which is the same as in our data. The rate of arterial hypertension with the need for vasodilator treatment was 20% in the review of Bagdure et al. [20], 11% in the ECPR-cohort, and only 2% in the present cohort. The rates of ICH presented in this cohort (18%) are comparable to other reported data sets (mostly derived from cohorts treated with VA-ECMO) [24], but higher than those in reported data from neonates with renal/urologic disease (13%) or neonates needing ECPR (14%). The reason for the markedly lower rate of CNS infarction (2%) in our data, as compared with the preterm infants in the study of Church et al. [24] of 18%, remains unclear. Various groups have reported a significantly higher rate of neurologic complications (e.g., hemorrhage, infarction, seizures, and brain death) in neonates treated with VA-ECMO [25, 26]. Furthermore, GA at birth was associated with a higher rate of neurologic complications. Although the GA of VA-ECMO-treated infants was lower in the present cohort, there was no significant difference regarding neurologic complication rates, neither individually nor combined.

### Limitations

Robustness of the present study might be biased by different influencing factors, including the retrospective design, and the selection and reporting bias due to the data collection via the ELSO registry. Furthermore, patient selection via ICD codes might lead to under- or overestimation of case numbers. Comparison between patient groups is difficult because these were not propensity matched, leading, for example, to more preterm babies in the VA-ECMO-treated group. Additionally, as in-house guidelines for neonatal ECMO treatment differ worldwide and between low- and high-volume centers, the comparison of registry-based data needs to be interpreted with caution. Nevertheless, the ELSO registry allows for the analysis of big cohorts and is essential for evaluation of ECMO indications and outcomes. As in other studies with this approach, there were no data available for follow-up after discharge and no information on long-term survival, neurologic development, and quality of life.

## Conclusion

This ELSO registry–based cohort study represents the first updated analysis of neonatal ECMO runs in neonates with a NIHF. Overall, ECMO is a potential lifesaving option in some of these critically ill neonates, despite facing high mortality and complication rates. ECMO should be performed in experienced quaternary referral centers due to the complexity of the disease and the required procedures. Preterm-born neonates are prone to higher complication rates and an adverse outcome. Therefore, treatment decisions should be made on an individual evaluation with close communication with the patients’ parents and with the attending medical team.

## Data Availability

No datasets were generated or analysed during the current study.

## Abbreviations

BW: Birth weight
CDH: Congenital diaphragmatic hernia
CNS: Central nervous system
ECLS: Extracorporeal life support
ECMO: Extracorporeal membrane oxygenation
ECPR: Extracorporeal cardiopulmonary resuscitation
ELSO: Extracorporeal life support organization
GA: Gestational age
ICD: International classification of diseases
ICH: Intracranial hemorrhage
IHF: Immunologically mediated hydrops fetalis
IQR: Inter-quartile range
NIHF: Non-immune hydrops fetalis
PH: Pulmonary hypertension
RRT: Renal replacement therapy
SD: Standard deviation
STROBE: Strengthening the reporting of observational studies in epidemiology
TTTS: Twin-twin-transfusion syndrome
VA: Veno-arterial
VV: Veno-venous
VVA: Veno-veno arterial
